# Supplementary material for: Splice-Junction-Based Mapping of Alternative Isoforms in the Human Proteome
Source: Cell Rep. Author manuscript; Available in PMC 2020 Jan 15. (PMC6961840; doi:10.1016/j.celrep.2019.11.026)
Supplement: 2 [file NIHMS1546469-supplement-2.zip › DF1/PXD006675/LeftAtrium/LeftAtrium_39_GPAA1_GTSADLTGHGTGPLCPASAGPTR.pdf]

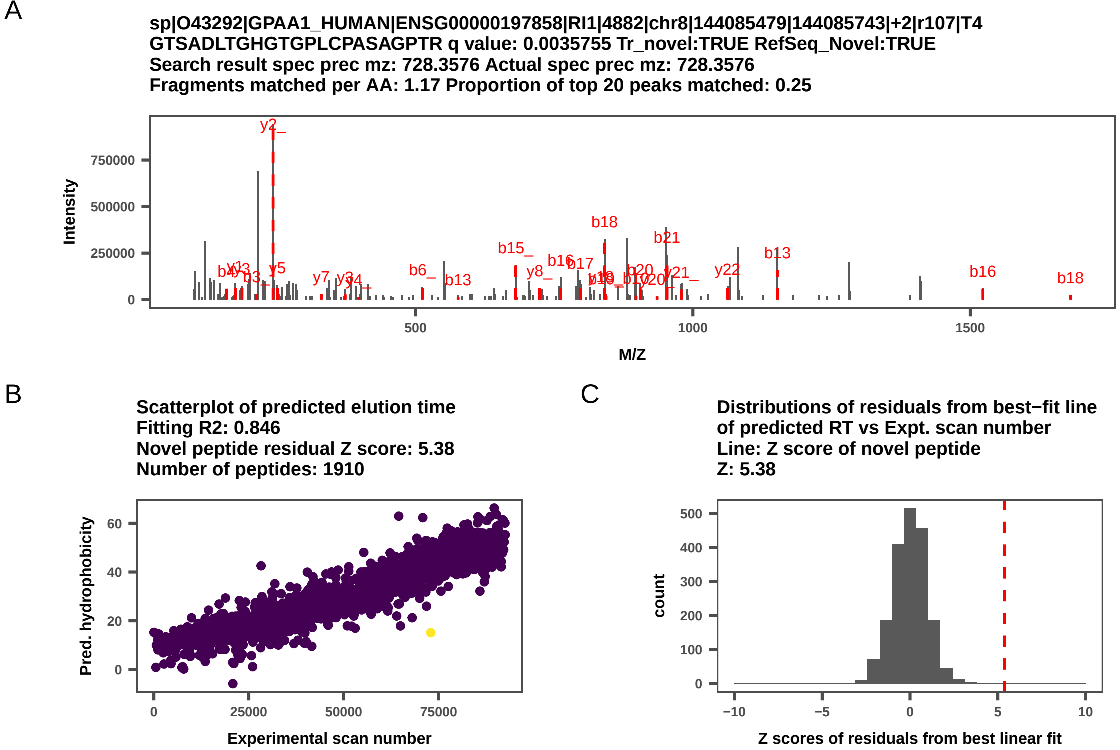
A
sp|O43292|GPAA1_HUMAN|ENSG00000197858|RI1|4882|chr8|144085479|144085743|+2|r107|T4
GTSADLTGHGTGPLCPASAGPTR q value: 0.0035755 Tr_novel:TRUE RefSeq_Novel:TRUE
Search result spec prec mz: 728.3576 Actual spec prec mz: 728.3576
Fragments matched per AA: 1.17 Proportion of top 20 peaks matched: 0.25
Intensity
750000
500000
250000
0
M/Z
500
1000
1500
y2_
b18
b21
b13
b15_
b16
b17
y8_
b6_
b13
y22
y21_
b16
b18
y7
y5
B
Scatterplot of predicted elution time
Fitting R2: 0.846
Novel peptide residual Z score: 5.38
Number of peptides: 1910
Pred. hydrophobicity
60
40
20
0
Experimental scan number
0
25000
50000
75000
C
Distributions of residuals from best−fit line
of predicted RT vs Expt. scan number
Line: Z score of novel peptide
Z: 5.38
count
500
400
300
200
100
0
Z scores of residuals from best linear fit
−10
−5
0
5
10
